# New concepts and challenges in the clinical translation of cancer preventive therapies: the role of pharmacodynamic biomarkers

**DOI:** 10.3332/ecancer.2015.601

**Published:** 2015-11-24

**Authors:** Karen Brown, Alessandro Rufini

**Affiliations:** Cancer Chemoprevention Group, Department of Cancer Studies, University of Leicester, Leicester LE2 7LX, UK

**Keywords:** therapeutic cancer prevention, pharmacodynamic biomarkers, translation, resveratrol, pharmacokinetics, curcumin, window trial

## Abstract

Implementation of therapeutic cancer prevention strategies has enormous potential for reducing cancer incidence and related mortality. Trials of drugs including tamoxifen and aspirin have led the way in demonstrating proof-of-principle that prevention of breast and colorectal cancer is feasible. Many other compounds ranging from drugs in widespread use for various indications, including metformin, bisphosphonates, and vitamin D, to dietary agents such as the phytochemicals resveratrol and curcumin, show preventive activity against several cancers in preclinical models. Notwithstanding the wealth of opportunities, major challenges have hindered the development process and only a handful of therapies are currently approved for cancer risk reduction. One of the major obstacles to successful clinical translation of promising preventive agents is a lack of pharmacodynamic biomarkers to provide an early read out of biological activity in humans and for optimising doses to take into large scale randomised clinical trials. A further confounding factor is a lack of consideration of clinical pharmacokinetics in the design of preclinical experiments, meaning results are frequently reported from studies that use irrelevant or unachievable concentrations. This article focuses on recent findings from investigations with dietary-derived agents to illustrate how a thorough understanding of the mechanisms of action, using models that mimic the clinical scenario, together with the development of compound-specific accompanying pharmacodynamic biomarkers could accelerate the developmental pipeline for preventive agents and maximise the chances of success in future clinical trials. Moreover, the concept of a bell-shaped dose-response curve for therapeutic cancer prevention is discussed, along with the need to rethink the traditional ‘more is better’ approach for dose selection.

## Introduction

Epidemiology studies and clinical trials of tamoxifen and aspirin highlight the enormous potential of therapeutic prevention for reducing cancer incidence and related mortality [1]. There is now compelling evidence that regular aspirin use is protective against the development of colorectal cancer (CRC) and subsequent metastatic disease, as well as recurrence after diagnosis [2, 3]. Similarly, it is well established that tamoxifen reduces the occurrence of oestrogen receptor-positive invasive breast cancer by ~43% and five years of treatment provides long-lasting protection for at least two decades [4–6]. The last decade has seen exciting developments in the field with accumulating reports suggesting various drugs in widespread use for other indications including metformin, bisphosphonates, and vitamin D may have protective effects against multiple cancers [7, 8].

Despite these exciting times, progress in the field to date has been slow, with only a handful of therapies currently approved for cancer risk reduction [9]. In contrast, the use of preventive strategies in cardiovascular medicine is routine practice and has contributed to a fall in heart disease and associated deaths over the past 30 years; indeed it has been estimated that early pharmacologic intervention to interrupt the atherogenic process accounts for ~50% of the decline in mortality rates [9]. A large part of the reason that cancer is lagging behind are several major challenges in the development process for ‘new’ preventive agents.

Cancer typically takes decades to develop; therefore, obtaining definitive evidence of preventive efficacy requires long-term, large scale clinical trials, which are extremely costly. Although such randomised trials have been performed in the past, including for primary cancer prevention in the general population, the outcomes have been largely disappointing, particularly for dietary supplements [1, 10–12]. Moving forward, it is clearly no longer feasible to embark on large scale trials in any of the prevention paradigms (primary, secondary, tertiary) without considerable clinical data demonstrating that the agent of interest, at the dose employed, has biological activity consistent with a protective anticancer effect ideally in the target tissue. It will also be important to gain an understanding of which individuals are most likely to experience a net benefit from a particular therapeutic intervention at an early stage of preclinical/clinical development. Differences in an individual’s response could be because of factors including genetic/epigenetic profiles, lifestyle and health status, as well as the genetic make-up of any cancer that develops. Therefore, mechanistically-informed biomarkers are required to distinguish the most appropriate people for inclusion in trials. The accurate identification of high-risk individuals for enrolment in trials would lead to more efficient, less expensive studies, of shorter duration and smaller size, with ability to retain sufficient power to demonstrate efficacy. This is also an area of intense interest, but is beyond the scope of this article.

One of the major obstacles to successful translation of promising laboratory findings to the clinic is a lack of biomarkers that provide an early read out of biological activity, akin to the measurement of blood pressure and cholesterol levels in response to cardiovascular drugs. Such pharmacodynamic (PD) biomarkers are needed for optimising doses to take into subsequent large clinical trials, and to generate enough evidence of desired biological activity in humans to provide a basis for a decision on whether an agent warrants further development. PD biomarkers may also serve as early predictors of efficacy, prior to the development of overt disease. A further confounding factor in the field is that there is often little consideration of clinical pharmacokinetics (PK) in the design of preclinical experiments, meaning results are frequently reported from studies where irrelevant or unachievable concentrations/doses are used.

**Figure 1.**
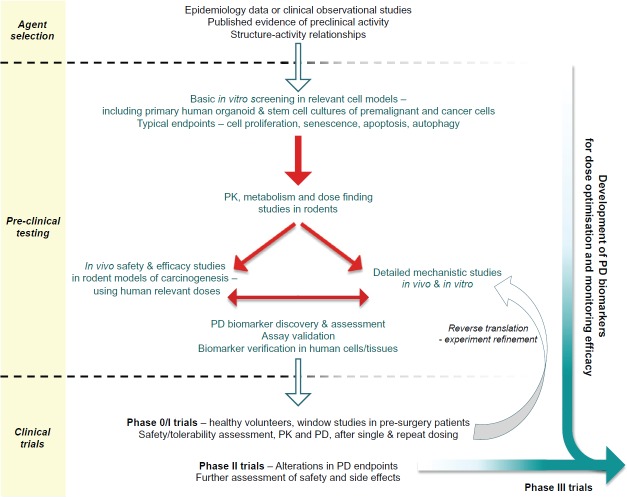
Proposed pathway for the preclinical development and early clinical evaluation of potential cancer prevention therapies.

This article focusses on the general development pathway for preventive agents (Figure 1) and highlights key areas relating to the discovery of PD or predictive biomarkers that warrant greater consideration in order to maximise the chances of success in future randomised controlled clinical trials.

## Importance of PD biomarkers

There are three broad approaches to the clinical use of agents for cancer prevention [1], although the definitions can vary and some researchers combine the first two scenarios under the term primary. ‘Primary therapeutic prevention’ involves the administration of compounds to the general population or those with particular risk factors, for example, those with a genetic predisposition (most notably familial adenomatous polyposis and Lynch syndrome in colorectal cancer) or those with exposure to a known carcinogen such as aflatoxin or asbestos. ‘Secondary prevention’ concerns individuals with precursor lesions such as colorectal adenomas, and aims to prevent progression to invasive cancer, whilst ‘tertiary prevention’ is defined as the administration of agents to prevent recurrence or second primary cancers in individuals who have undergone successful treatment of earlier disease. In all three scenarios, the subjects are essentially ‘cancer-free’; in most cases they are not guaranteed to progress or relapse and are unlikely to have clinically detectable malignant disease, or display any associated biomarkers, at least at the start of any prevention trial. Consequently, the initial stages of clinical evaluation for any new agent must rely on PD biomarkers for dose titration to identify the optimal range, and for evidence that the agent is hitting its intended target(s) and having the desired downstream effects.

The discovery and development of agents should be conducted in parallel with the search for a panel of accompanying PD biomarkers. This should allow sufficient time for selection of the best candidates and for the development and validation of suitable assay methods (to standards of Good Clinical Practice/Good Clinical Laboratory Practice), prior to the design of clinical trial protocols. PD biomarkers may be generic indicators of a process such as apoptosis or cell proliferation or more specific measures at a molecular or biochemical level relating to a known (or anticipated) mechanism of action. In both cases, there should be convincing preclinical data, including evidence from an appropriate *in vivo* model that biomarker changes correlate with efficacy.

Pre-surgical window of opportunity trials, which take advantage of the interval between a diagnosis of cancer and surgical resection to administer a preventive agent, present a powerful platform for evaluating the PD effects of an agent in its proposed target tissue [13]. This approach has been successfully employed in a variety of trials across several solid malignancies, sometimes in the phase 0 setting, which is a first in human trial without therapeutic or diagnostic intent [14]. Short-term lapatinib given prior to surgery was found to decrease cell proliferation, quantified by Ki-67 labelling index, in HER-2-positive breast cancer tissue and premalignant ductal intraepithelial neoplasia, supporting further exploration of this drug for breast cancer prevention in high-risk patients [15]. Pharmacologically active concentrations of curcumin, a constituent of turmeric, were shown to reach the colorectum of patients who took 3.6 g daily during the week leading up to surgery. Activity was reflected by a significant reduction in the levels of a deoxyribonucleic acid (DNA) damage marker of oxidative stress in malignant tissue after curcumin therapy [16]. However, it should be recognised that it may be difficult to directly compare measurement of some biomarkers in pre-intervention biopsy samples with post-intervention surgical tissues taken from the same patient. Technical differences in the procedure may themselves influence biomarker levels and there is a potential for sampling errors because of the small amounts of tissue available at biopsy [17]. These possibilities emphasise the importance of incorporating a placebo or control group into biomarker trials to fully appreciate the influence of procedural and technical factors.

In some cases it may be possible to obtain multiple sequential biopsy samples from the same person, to ascertain the effect of a preventive therapy. These studies may be performed in healthy volunteers and the samples taken are often normal tissue, although the amount of material available for analysis is a major limitation. This approach has been used to determine the lowest dose of aspirin capable of significantly suppressing prostaglandin E2 and F2α concentrations in colorectal tissue [18] and to demonstrate that oral ingestion of aspirin can modulate effectors of the mechanistic target of rapamycin (mTOR) in normal human rectal mucosa [19].

To date, generic biomarkers of cell proliferation or apoptosis have been most commonly evaluated in tissues obtained from window trials and there are fewer reports demonstrating the successful analysis and modulation of a specific target or pathway in tissue after intervention [20–22]. This may be because virtually all of the compounds under consideration, whether they be of synthetic or natural origin, engage multiple mechanisms rather than hitting a solitary target and their fundamental modes of action for prevention of cancer in humans have not yet been fully elucidated. While this multi-targeted behaviour is a distinct advantage for interfering with the numerous processes that contribute to cancer development, it makes it difficult to delineate the key effects versus those that are collateral. Although the task is challenging, it is imperative that a greater understanding of the mechanisms of action is obtained for every promising agent, as this information will ultimately be required to identify mechanistically-rational biomarkers to help define those people most likely to benefit and to demonstrate meaningful biological activity in early phase trials.

Aberrations in DNA methylation deregulate the genome and contribute to the loss of tissue homeostasis observed in cancer. Since they are potentially reversible, epigenetic alterations represent promising targets for cancer prevention therapies. Accordingly, the analysis of epigenetic changes, particularly genome-wide DNA methylation patterns, is receiving considerable attention for the discovery of tissue and blood-based biomarkers of cancer risk and also as indicators of biological activity or efficacy of preventive interventions [23, 24]. Recently, the use of aspirin has been associated with modulation of age and cancer-related DNA methylation changes in normal colonic epithelium of women, suggesting it can impact the evolution of cancer methylomes [24].

In addition to tissue biomarkers, there is also a need for more readily accessible indicators of PD effects that are amenable to repeated measurement and application to larger scale trials for monitoring activity and predicting efficacy over time. Being minimally-invasive, plasma represents an ideal resource for biomarker discovery and analysis as it contains high concentrations of proteins, metabolites, and nucleic acids whose modulation might reflect activity of the preventive agent in the target tissue [25–27]. However, specific effects detectable in a target tissue may be substantially diluted, leading to reduced sensitivity. A further advantage of plasma-based biomarkers is that they offer the opportunity to characterise biomarker kinetics, which may be important if therapy-induced changes are short lived or modulated by factors such as diurnal variation [28]. It can be difficult, if not impossible, to control for kinetic influences in the surgical setting, particularly in large scale trials. Ideally, any surrogate biomarkers should be linked to the mechanism of action, but at the very least they must be shown to correlate with efficacy in preclinical models.

## Improving the relevance of preclinical models

The first stage of development for prospective cancer preventive agents has traditionally involved basic screening for antiproliferative and pro-apoptotic activity in cell lines, with a view to prioritising compounds for efficacy evaluation *in vivo* and more detailed mechanistic interrogation. Most publications report the use of cancer cell lines for this purpose, exposed just once to concentrations that are often considerably higher than those that could ever be achieved in human plasma/tissues. This approach may highlight mechanisms of action that turn out to be irrelevant in humans or lead to inaccurate predictions of clinical efficacy. Discrepancies between preclinical testing paradigms and the eventual clinical scenario may partly explain the disappointing outcome of some randomised chemoprevention trials [12].****

### Importance of clinical PK

Reverse translation of PK data from phase I trials back into the laboratory should be an essential component of the development process for any agent being newly considered for cancer prevention. Remarkably, for many diet-derived compounds under intense preclinical study this information is not known, and while the plasma PK are normally well characterised for repurposed drugs, the concentrations reaching potential target tissues will not usually have been investigated. This information could have a major bearing on whether a therapy ultimately advances to further clinical testing because it will impact on the quality and potential human relevance of the supporting preclinical data package. In this respect, it can define the upper boundary for concentrations and doses that should be used in preclinical studies, according to the maximum acceptable daily dose based on toxicity and tolerability data and/or the maximum number of capsules that can be taken without compromising patient compliance. For example, although curcumin is both poorly absorbed and rapidly metabolised after oral ingestion, a pilot study involving patients undergoing colorectal endoscopy or surgical resection revealed prolonged biologically active colonic tissue levels of the parent compound in biopsies from all participants up to 40 hours after the last dose [29]. This contrasts with the situation in plasma where curcumin was only quantifiable in samples from one fifth of the patients and concentrations were ~1000-fold lower. Whilst these findings support the continued investigation of curcumin for the prevention of colorectal malignancies they do not rule out pursuing curcumin in the management of other cancers, rather they suggest that the activity of low, systemically achievable concentrations should be explored in studies involving organs distant to the gastrointestinal tract.

Recently, patient derived xenografts (PDX) have attracted much interest for drug development studies, where they can reproduce the complex heterogeneity and diversity of human cancers. Despite technical challenges rendering the generation of PDX models from pre-malignant tissues difficult, PDXs derived from more advanced malignancies might still prove a valuable tool for investigating PD or mechanistic biomarkers in human tissue within pre-clinical studies [30]. In this context they may also be useful for identifying the most suitable and robust analysis methods for translation to clinical trials, with the caveat that PK factors may be less clinically relevant if the tumour is grown subcutaneously rather than orthotopically.

### Role of metabolites

Clinical PK studies may also highlight interesting metabolites worthy of investigation. Extensive metabolism leading to poor systemic bioavailability is a perceived limitation of many dietary phytochemicals. However, it is possible that certain metabolites may actually contribute to, rather than diminish efficacy, by virtue of either intrinsic activity or an ability to regenerate the parent compound. This was recently found to be the case for one of the major human metabolites of resveratrol [31], where its sulfate conjugates were demonstrated to undergo hydrolysis *in vivo*, liberating free resveratrol in target tissues and plasma of mice. Conversion to the parent also occurred in human colorectal cancer but not in normal cells, with the extent of cellular uptake being dependent on specific membrane transporters, including organic anion–transporting polypeptide 1B3. When cells were exposed to clinically achievable concentrations of resveratrol sulfates the extent of anti-proliferative activity was dictated by the intracellular levels and was mediated by the induction of autophagy and senescence. These effects were abrogated by inclusion of a sulfatase inhibitor which reduced intracellular resveratrol production. These observations suggest that resveratrol may be delivered to target tissues in a stable sulfate-conjugated form where the parent compound is gradually regenerated in selected cells and can exert effects consistent with cancer prevention, encouraging its further use in human trials. Consideration may however need to be given to the profile of membrane transporters present in each potential target organ and in normal tissue versus premalignant or cancer, as this could influence efficacy.

### Developing preventive in vitro models

An additional challenge in therapeutic cancer prevention is selecting the most appropriate cellular model for *in vitro* studies. Immortalised ‘normal’ and cancer cell lines have proven valuable to date for identifying mechanisms of action. However, in the prevention setting it is unlikely that these will actually correspond to the target cell population in humans, particularly if the interventions begin early enough. Premalignant cells would provide a better system and indeed mimic a commonly employed clinical model for prevention trials, such as women with breast intraepithelial neoplasia [32] and people who have had colorectal adenomas removed and hence are at increased risk of recurrence or progression [33]. However, to date such cells have been difficult to acquire and few if any are commercially available, but with technological advances in 3D-organoid culture it is possible to establish and maintain long term cultures of primary human normal, adenoma, and cancer cells obtained from colonic mucosa of patients [34]. Analogous methods have been established for other tissues including pancreas, stomach, and liver [35]. Although primary organoids are more challenging to handle and more expensive to use than immortalised cell lines, they present an excellent opportunity to screen agents in cells that represent the earliest stages of the carcinogenic pathway and reflect the heterogeneity present in humans, plus the different cancer subtypes [34, 36]. As a promising new technology that is only just being employed for the assessment of cancer treatments, work is needed to ascertain how well patient-derived organoids are able to accurately predict clinical response to preventive therapies.

Another valuable platform for validating mechanisms of action and candidate biomarkers are explant cultures i.e. using thin sections of human tissue. These have the added advantage of conserving the full cellular and microenvironmental heterogeneity plus 3D-architecture [37, 38]. Furthermore, considering that the amount of precious tissue from therapeutic prevention trials is inevitably limited, explants taken directly from untreated control patients or from a PDX exposed *ex vivo* to the agent of interest can be used for pre-selecting the most robust biomarkers and optimising the analysis methods, before application to trial samples. Such an approach has been used to verify that the ability of low dose resveratrol to activate AMP-activated protein kinase (AMPK) signalling and cause autophagy in mouse models translates to human tissue [39].

A further consideration in designing *in vitro* or *ex vivo* experiments is the dosing strategy. The measurable effects of preventive agents may be relatively subtle, especially when low clinically attainable concentrations are employed. In fact, such low concentrations may be ignored by researchers because of a lack of activity using standard single-dose treatments, but it is possible that important effects may only become evident following a repeat dose protocol over several days or weeks [40]. The need to tailor experiments and endpoints specifically to a prevention paradigm is reinforced by the inherent differences between cytotoxic anticancer drugs for treatment versus preventive agents. The former aim to kill cells upon acute dosing, while prevention may require sustained interaction between the agent and target tissues, and there may only be a small impact on gross cell number in culture-based assays.

To mimic clinical scheduling and take account of any degradation/metabolism, it is worth replenishing the media plus preventive agent on a daily basis. This type of repeat dose protocol was used over six days to demonstrate that dietary achievable concentrations of resveratrol, as low as 10 nM, have cancer preventive activity in colorectal adenoma cells [39]. Rather than apoptosis, which is commonly reported at concentrations exceeding ~10 μM [41, 42], resveratrol-induced autophagy as a short-term response, whilst senescence appeared to be the result of sustained exposure to low resveratrol concentrations [39]. This study highlights the potential contribution of autophagy and senescence to cancer prevention as a means of inhibiting the proliferation of aberrant cells [43, 44] and demonstrates the importance of chronic exposure for assessment of preventive activity.

## Dose selection for clinical trials with dietary-derived agents

In the context of cancer prevention the safety of any therapy is absolutely paramount. This means that if agents are to be translated to the clinic in a timely manner the choice is restricted to dietary-derived compounds that are already consumed regularly by human populations and existing drugs that have been in widespread use for other chronic indications and have suitable safety profiles for repurposing [45].

Dietary agents are the focus of intense interest internationally; however, despite extensive preclinical data indicating that phytochemicals, micronutrients, and vitamins can protect against various cancers, these findings have failed to translate into successful outcomes in randomised controlled trials and in some cases cancer incidence has actually increased in the intervention group [10, 46]. These unforeseen results have been partly attributed to a failure to identify an optimal dose before embarking on large costly trials. It has been suggested by some investigators involved in the β-Carotene and Retinol Efficacy Trial that use of an inappropriately high dose of β-Carotene may explain the increase in lung cancer that was observed in smokers [11, 46].

To date, little attention has been paid to the fundamental issue of how to determine appropriate doses for translation to clinical prevention studies, and instead the classic drug development paradigm has been embraced—that ‘more is better’. This approach is at odds with the epidemiology data that often first alert researchers to the possibility that a dietary constituent might be protective against cancer. Such observations are typically founded on populations consuming low amounts of a substance over a long period [47, 48]. This would suggest that dietary achievable concentrations should be a focus of scientific interest, but virtually nothing is known about the PK or activity of such low exposures for any of the commonly investigated agents.

A recent study set out to challenge the assumption that ‘more is better’ in the context of cancer prevention, using resveratrol as a model phytochemical [39]. In keeping with many other diet-derived agents, resveratrol has been touted as worthy of clinical evaluation, but significant knowledge gaps, namely the key molecular targets in humans and identification of the optimal dose, prevent the rational design of trials to assess efficacy. To address these deficiencies the target tissue distribution and activity was compared for a low dietary-relevant dose of resveratrol, i.e. equivalent to the amount contained in a large glass of red wine (5 mg) [49] and an intake 200-times greater (1 g). The higher dose has previously been used in phase I clinical trials [21, 50] and is considered the maximum that can be taken chronically by healthy populations, because of an increased risk of gastrointestinal side-effects at doses exceeding this level [51]. Initially, the dose response relationship and metabolite profile of [^14^C]-resveratrol was established in colorectal tissue of patients participating in a pre-surgical window trial. Importantly, the results proved that a dietary dose of resveratrol could reach its purported target tissue (colorectal mucosa) and defined the full achievable concentration range in humans. Daily administration of the equivalent doses to *Apc^Min^* mice (which represent a model of human colorectal carcinogenesis), revealed that the low dose inhibited intestinal adenoma development with much greater potency than the higher dose. Surprisingly, this phenomenon only occurred in animals maintained on a high-fat diet; when the mice were fed a standard-fat diet the low dose was completely ineffective. Efficacy was highly correlated with increased expression and activation of the energy sensor AMPK in mouse intestinal mucosa, and was associated with the induction of autophagy and senescence, plus reduced cell proliferation in adenomas. Mechanistic studies using adenoma cells derived from an *Apc^Min^* mouse revealed a bell-shaped dose response for components of the AMPK signalling pathway, culminating in mTOR inhibition, and the induction of autophagy and senescence at low concentrations. AMPK activation appeared to be mediated via both reduction of the ATP/AMP ratio and increased production of reactive oxygen species. The pro-oxidant effect of low dose resveratrol was also evident in colorectal tissue of patients participating in the window trial; those taking 5 mg resveratrol daily had significantly higher levels of oxidative stress markers compared to those taking 1 g daily and a control population. Taken together these findings illustrate that low dietary exposures of resveratrol not only elicit biological changes in mouse and human tissues pertinent to colorectal cancer prevention, but they have greater efficacy compared to high doses. The same may be true for some other diet-derived agents; therefore, it is worth expanding preclinical testing strategies to include a dose range that encompasses levels achievable through both dietary intake and pharmacological therapy.

Further support for a rethinking of translational approaches is provided by results from trials such as SELECT, where contrary to the original hypothesis, supplementation with selenium increased the risk of high-grade prostate cancer among men with high baseline selenium stores, and supplementation with vitamin E increased the risk among individuals with low selenium stores at baseline; neither intervention was protective [10]. In addition, a U-shaped dose response has been described for vitamin D and breast cancer risk and is also complicated by basal circulating levels of this vitamin [52].

The existence of unexpected non-linear dose-response relationships emphasises the absolute requirement for robust PD biomarkers, which have been shown in preclinical models to correlate with efficacy for a particular therapy. The biomarkers are critical for accurately defining the human dose-response relationship in early phase trials, in order to select the optimal dosing regimen, prior to embarking on phase III trials.

### Diet and metabolic status as interacting factors

The requirement for a high-fat diet to expose the efficacy of low dose resveratrol [39] highlights the potential for interactions between cancer preventive therapies and other components of the diet or lifestyle factors, which may be important when identifying patient populations for inclusion in trials. Feeding mice a high-fat diet is a commonly used model of impaired glucose intolerance and early type 2 diabetes [53], which is a risk factor for human colorectal cancer [54, 55]. Although the mechanisms underlying the reported interaction are not currently known, the findings are consistent with results emerging from other clinical trials exploring the metabolic effects of resveratrol, where it appears to have selective activity in obese humans [56] or those with pre-existing metabolic disorders [57, 58]. It is notable that several other prominent candidates for cancer prevention, including aspirin and metformin also cite activation of AMPK signalling among their numerous mechanisms of action [19, 59–61]. It is therefore conceivable that whether an individual responds to all these therapies may be influenced by their metabolic status and/or diet/lifestyle, which illustrates the importance of working towards personalised preventive therapy. In fact, a recent study has shown that at conventional antidiabetic doses the effect of metformin on tumour Ki-67 indices in a non-diabetic breast cancer patients varies with tumour and host characteristics, particularly those relating to insulin resistance [62]. More preclinical research is needed to understand the mechanisms underpinning any interactions between diet/metabolic status and efficacy of preventive therapies, as very little is known about the subject. These host variables need to be taken into account early in the evaluation of new agents which may necessitate the identification of suitable metabolic biomarkers for incorporation into animal efficacy studies and clinical trials.

## Conclusion

In recent decades there has been encouraging progress in the field of therapeutic cancer prevention but the enormous potential of this strategy is still largely untapped. Its importance is underlined even further by emerging data that demonstrate the complex and heterogeneous genomic landscape in cancer [63]. Given the degree of heterogeneity and the rapidity of ensuing resistance, treatment of advanced disease by targeting driver aberrations represents a massive challenge that may yield limited success in terms of cure [64]. The complexity of malignant disease provides impetus for alternative approaches. In this respect, targeting cells at the premalignant stage in a prevention setting, when they should theoretically contain fewer genetic alterations, and before the emergence of widespread heterogeneity and massive genomic instability presents an exciting opportunity [65].

There is a wealth of dietary and pharmacological compounds that show convincing cancer preventive activity in laboratory models with potential for clinical translation. However, the failure of previous large and expensive trials has outlined the necessity to develop (**i**) a better understanding of the key mechanisms responsible for efficacy, (**ii**) a detailed knowledge of the *in vivo* PK, (**iii**) to delineate the dose-response relationships for PD changes over a range relevant to humans, and (**iv**) to identify robust biomarkers that convincingly mirror therapeutic efficacy, before costly, time-consuming randomised clinical trials are initiated. Another integral component to maximise successful cancer prevention will be the identification of high-risk populations as well as individuals that are more likely to benefit from intervention. Indeed, it is likely that the efficacy of preventive compounds will vary based on cancer genetic profile, and in the era of precision medicine information on host and tumour genetic/epigenetic characteristics will also inevitably shape what must be the ultimate longer term ambition—personalised preventive therapy. Advances in technological platforms, such as next generation sequencing and digital polymerase chain reaction (PCR) for the sensitive detection of circulating free tumour DNA [66], should increase the chances of delivering biomarkers for the detection of early or premalignant disease to reliably identify high-risk populations for enrolment in trials.

In summary, developing safe and efficacious cancer preventive therapies that are well-characterised mechanistically, and have accompanying PD biomarkers, together with an effort to integrate precision medicine and cancer genomics are priority research areas that have potential for major clinical impact.

## Conflicts of interest

The authors declare that they have no conflict of interest.
